# Arachidonic acid inhibition of the NLRP3 inflammasome is a mechanism to explain the anti-inflammatory effects of fasting

**DOI:** 10.1016/j.celrep.2024.113700

**Published:** 2024-01-23

**Authors:** Milton Pereira, Jonathan Liang, Joy Edwards-Hicks, Allison M. Meadows, Christine Hinz, Sonia Liggi, Matthias Hepprich, Jonathan M. Mudry, Kim Han, Julian L. Griffin, Iain Fraser, Michael N. Sack, Christoph Hess, Clare E. Bryant

**Affiliations:** 1 Department of Veterinary Medicine, University of Cambridge, Cambridge, UK; 2 Signaling Systems Section, Laboratory of Immune System Biology, National Institute of Allergy and Infectious Disease (NIAID), National Institutes of Health (NIH), Bethesda, MD, USA; 3 The Cambridge Institute of Therapeutic Immunology and Infectious Disease (CITIID), University of Cambridge, Cambridge, UK; 4 Laboratory of Mitochondrial Biology and Metabolism, National Heart, Lung and Blood Institute (NHLBI), NIH, Bethesda, MD, USA; 5 Department of Biochemistry, University of Cambridge, Cambridge, UK; 6 University Hospital Basel, Basel, Switzerland; 7 Cantonal Hospital of Freiburg, Freiburg, Switzerland; 8 Department of Medicine, University of Cambridge, Cambridge, UK; 9 Present address: Division of Infectious Diseases and Immunology, Department of Medicine, University of Massachusetts Chan Medical School, Worcester, MA, USA; 10 Present address: Shimadzu UK Ltd., Milton Keynes, UK; 11 Present address: Healx Ltd., Cambridge, UK; 12 Present address: The Rowett Institute, University of Aberdeen, Aberdeen, UK; 13 These authors contributed equally; 14 Lead contact

## Abstract

Elevated interleukin (IL)-1β levels, NLRP3 inflammasome activity, and systemic inflammation are hallmarks of chronic metabolic inflammatory syndromes, but the mechanistic basis for this is unclear. Here, we show that levels of plasma IL-1β are lower in fasting compared to fed subjects, while the lipid arachidonic acid (AA) is elevated. Lipid profiling of NLRP3-stimulated mouse macrophages shows enhanced AA production and an NLRP3-dependent eicosanoid signature. Inhibition of cyclooxygenase by nonsteroidal anti-inflammatory drugs decreases eicosanoid, but not AA, production. It also reduces both IL-1β and IL-18 production in response to NLRP3 activation. AA inhibits NLRP3 inflammasome activity in human and mouse macrophages. Mechanistically, AA inhibits phospholipase C activity to reduce JNK1 stimulation and hence NLRP3 activity. These data show that AA is an important physiological regulator of the NLRP3 inflammasome and explains why fasting reduces systemic inflammation and also suggests a mechanism to explain how nonsteroidal anti-inflammatory drugs work.

## In brief

By studying how arachidonic acid (AA) impacts the inflammasome, Pereira et al. demonstrate that AA inhibits NLRP3-mediated IL-1β production by blocking the activities of phospholipase C and the downstream protein kinases PKD and JNK. This provides a mechanistic basis for the inverse relationship between serum levels of AA and IL-1β.

## Graphical Abstract

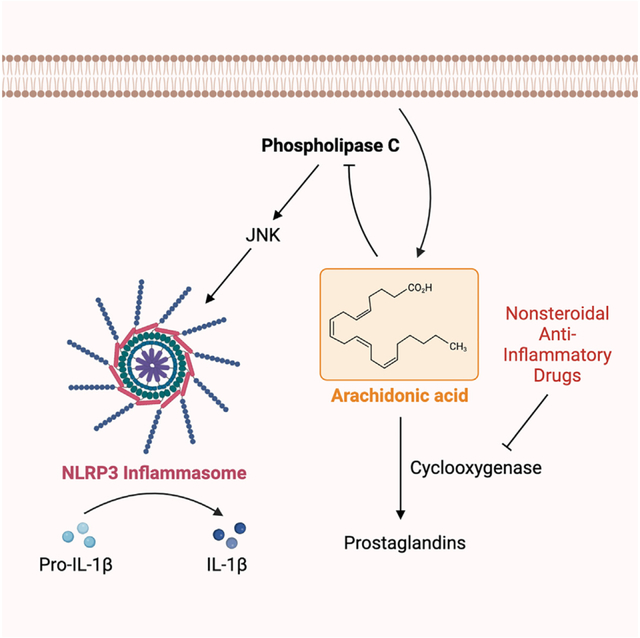

## INTRODUCTION

The consumption of a Western, high-calorie diet (WD) is associated with a chronic metabolic inflammatory syndrome (metaflammation), which underpins many prevalent noncommunicable diseases.^1^ How this complex process, whereby tissue-specific and systemic immune responses are integrated alongside metabolic regulation, is governed remains poorly understood. Fasting leads to suppression of metabolic inflammation and is characterized by a drop in serum pro-inflammatory cytokines, particularly interleukin-1β (IL-1β), which is closely associated with insulin regulation and blood glucose levels.^2–4^

One emerging regulator of metaflammation is the inflammasome induced by nucleotide-binding and oligomerization domain-like receptor (NLR) family pyrin domain-containing 3 (NLRP3) activation.^1^ Inflammasomes are multi-protein signaling platforms that consist of a receptor (usually an NLR), an adaptor (apoptosis-associated speck-like protein containing a caspase-recruitment domain, ASC), and an effector (caspase-1), which jointly process IL-1β and IL-18 to their bioactive forms and cleave the cell death effector gasdermin D (GSDMD) to drive pyroptosis.^5^ Fasting regulates NLRP3 activity,^2^ but the mechanisms that regulate this process are not well understood. Oxidized low-density lipoprotein (LDL) and cholesterol crystals trigger activation of NLRP3 in macrophages when the cellular capacity for metabolizing cholesterol is exceeded, but if the cell remains capable of processing cholesterol, then anti-inflammatory responses are induced.^1^ Diets rich in saturated fatty acids, such as palmitic acid (PA) or stearic acid, also trigger NLRP3 inflammatory activity.^1^ In mice, the systemic inflammation is seen when *Ldlr*^−/−^ are fed a WD and requires NLRP3-mediated trained immunity, which is reversed when the mice are placed on a normal chow diet.^6^

Activation of the NLRP3 inflammasome is a tightly regulated two-step process.^7^ First transcription of pro-IL-1β and NLRP3 is induced by Toll-like receptor (TLR) activators, such as lipopolysaccharide (LPS), or pro-inflammatory cytokines, such as tumor necrosis factor α. This is followed by an activation phase, for which many post-translational regulatory steps are important, one of which is phosphorylation of both NLRP3 and ASC.^7^ Phosphorylation of ASC by spleen tyrosine kinase and c-Jun N-terminal kinase (JNK) leads to the formation of the ASC signaling scaffold and to inflammasome activation.^8^ The regulation of NLRP3 by phosphorylation is more complex, with the pyrin domain of this protein being the phospho-regulatory target.^9^ We have shown that biphasic JNK1 phosphorylation of NLRP3 in response to mitochondrial reactive oxygen species leads to inflammasome activation,^10^ but phosphorylation of NLRP3 at serine 5^8^ and JNK2 activity both prevent activation of the NLRP3 inflammasome.^11^ This suggests there is a complex network of phosphorylation events that regulate NLRP3.

NLRP3 is increasingly recognized as being interlinked with lipid metabolism.^12–14^ Eicosanoids, which are derived from oxidation of arachidonic acid (AA), comprise a complex family of signaling lipids with important roles in regulating inflammation.^15^ Among them, prostaglandins (PGs), such as PGE_2_, formed by cyclooxygenase (COX) metabolism of AA, may regulate NLRP3 activity, although the precise role for this lipid as an activator or inhibitor of inflammasome activity is controversial.^16^ The nonsteroidal anti-inflammatory drugs (NSAIDs) are COX inhibitors, yet despite these medicines being in clinical use for decades, how they achieve their anti-inflammatory effects is still not fully understood.^17^

Here, we show that in fasting subjects where serum IL-1β is suppressed, AA is elevated, which is reversed upon refeeding. NLRP3 stimulation induced lipid production, including AA and eicosanoids, from macrophages, but only AA profoundly inhibited this inflammasome by suppressing phospholipase C (PLC) and JNK activity. NSAID treatment of macrophages suppressed NLRP3 activity and prostanoid production, but AA levels remained unchanged. These data show that AA is an important physiological regulator of the NLRP3 inflammasome and provide a mechanism explaining why fasting reduces systemic inflammation.

## RESULTS

### Fasting human subjects have elevated AA levels

**Figure 1.**
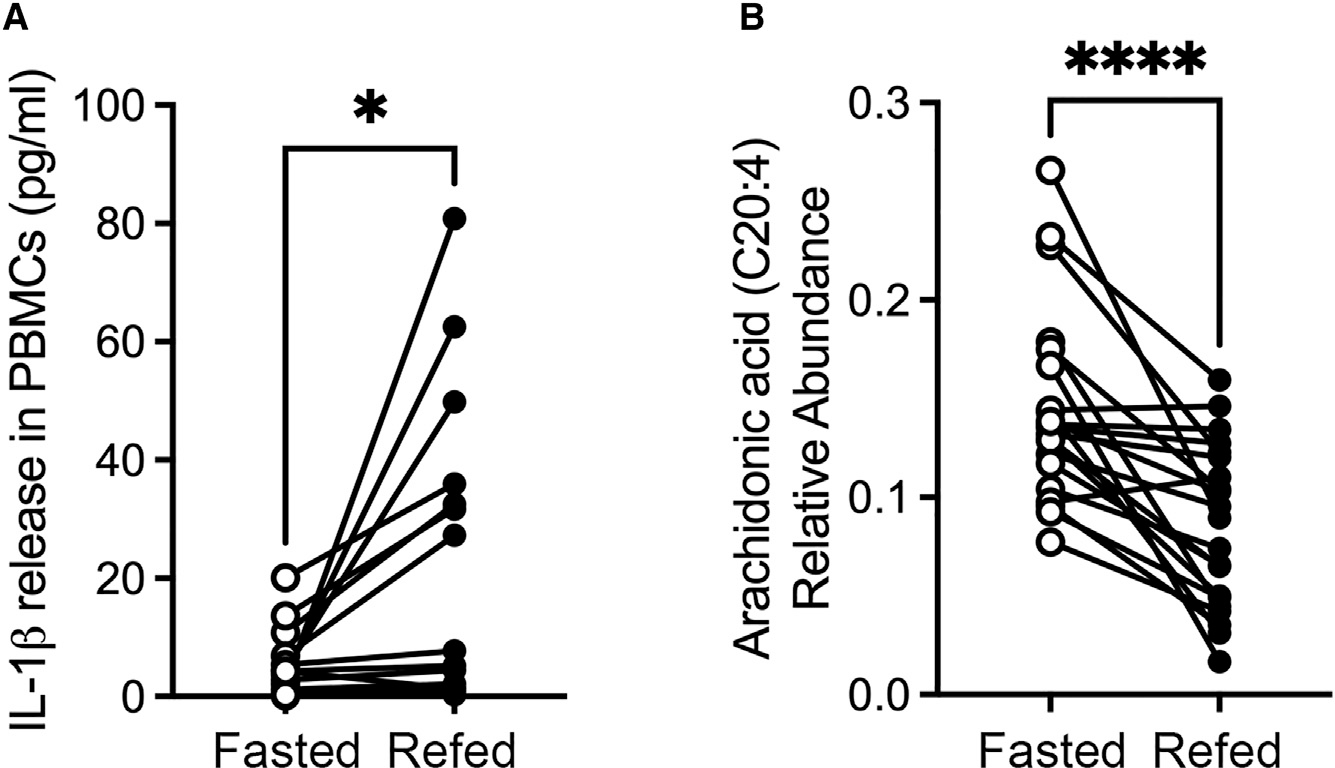
Fasting human volunteers have elevated arachidonic acid (AA) levels (A) Peripheral blood mononuclear cells were isolated from the volunteers and IL-1β production measured by ELISA before and after refeeding (n = 16). (B) Plasma AA was measured in the same volunteers before and after fasting (n = 21).

The emerging importance of lipids in regulating NLRP3 activity^12^ led us to investigate the lipidomic profile in fasted compared to fed individuals. Serum samples were taken from a cohort of volunteers. In this study, 21 volunteers consumed a baseline 500 kcal meal, fasted for 24 h, and then consumed a 500 kcal refeed meal. In peripheral blood mononuclear cells (PBMCs) from these volunteers, IL-1β levels were elevated 3 h after refeeding (Figure 1A). Plasma AA was elevated in the volunteers during fasting but reduced upon refeeding (Figure 1B).

### Canonical NLRP3 inflammasome activation leads to AA production

**Figure 2.**
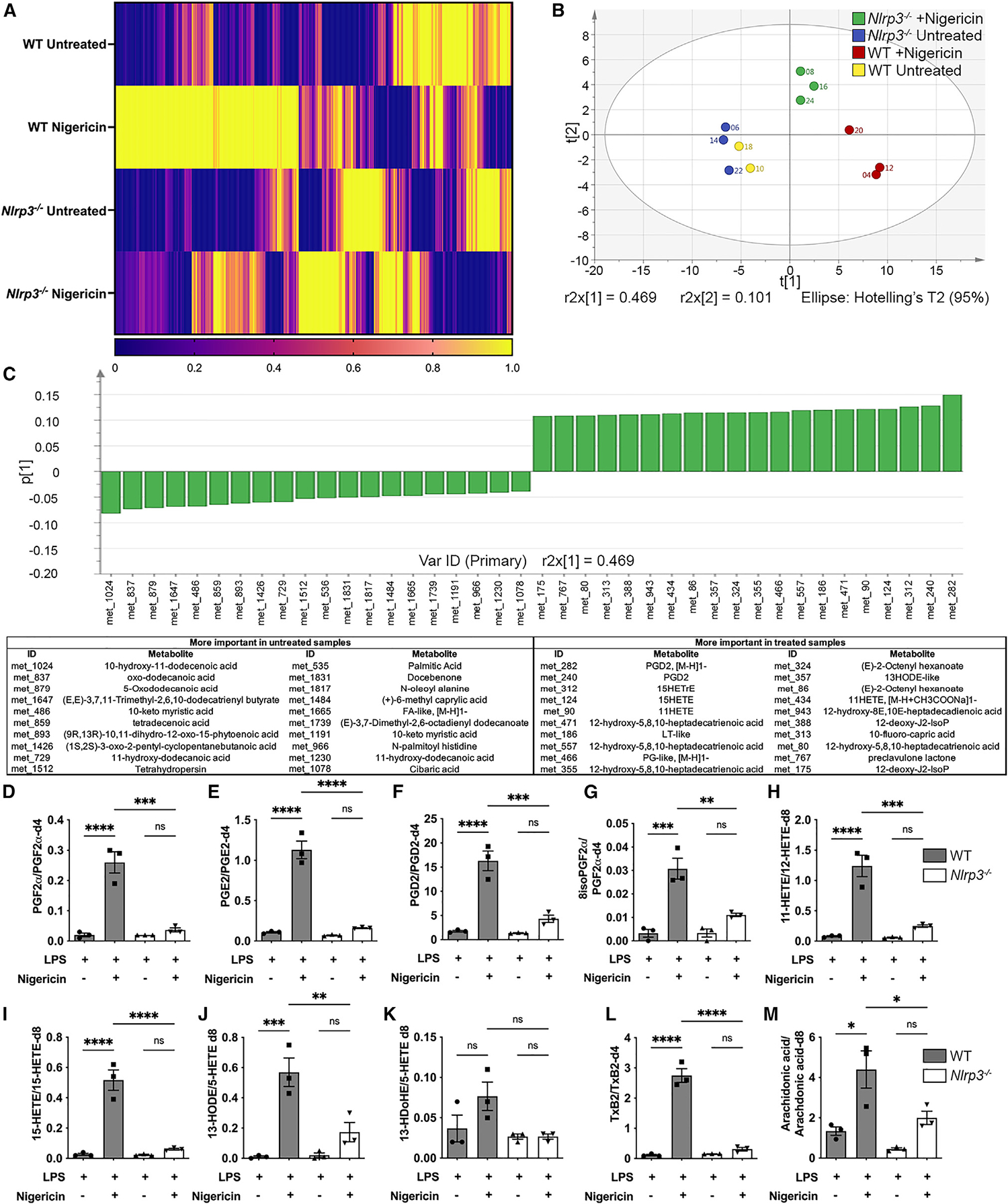
Canonical NLRP3 inflammasomes leads to eicosanoid production (A) Heatmap analysis of lipids present in culture supernatants of LPS-primed (200 ng/mL for 3 h) WT and *Nlrp3*^*−/−*^ BMDMs stimulated with nigericin (10 μM, 1 h), identified by liquid chromatography/drift tube ion mobility-mass spectrometry (LC/DTIM-MS). (B) Principal-component analysis of the samples used in this study. (C) Lipids that contribute the most to the differences between nigericin-treated and untreated groups. (D–M) Quantification by LC/DTIM-MS of eicosanoids PGF_2_α (D), PGE_2_ (E), PGD_2_ (F), 8-iso-PGF_2_α (G), 11-HETE (H), 15-HETE (I), 13-hydroxydocosahexaenoate (J), 13-hydroxyoctadecadienoic acid (K), TxB_2_ (L), and AA (M), present in culture supernatants of LPS-primed (200 ng/mL for 3 h) WT and *Nlrp3*^*−/−*^ BMDMs stimulated with nigericin (10 μM, 1 h). *p < 0.05, **p < 0.01, ***p < 0.001, ****p < 0.0001 (one-way analysis of variance with Tukey’s multiple comparison test). Data are from three independent experiments (mean and SEM).

The elevation of AA production coupled to the suppression of IL-1bβ production in fasted subjects led us to investigate how AA and its eicosanoid metabolites are synthesized upon NLRP3 stimulation with nigericin in macrophages. Wild-type (WT) and *Nlrp3*^−/−^ bone-marrow-derived macrophages (BMDMs) were primed with LPS (3 h, 200 ng/mL) and stimulated with nigericin (10 μM, 1 h). As expected, IL-1β production only occurred in nigericin-treated WT BMDMs (Figure S1). The culture supernatants were collected and lipids extracted and submitted to reverse-phase liquid chromatography/drift tube ion mobility-mass spectrometry.^18^ Identification was carried out using an internal database and LIPIDMAPS^19,20^ to match exact mass and collision cross-sections derived from the ion mobility measurements and then subjected to analysis.^21,22^ Heatmaps and principal-component analysis of the samples revealed clustering between primed WT and *Nlrp3*^−/−^ BMDMs, suggesting that the lipid compositions of these cell populations are similar. Upon nigericin stimulation, however, WT and *Nlrp3*^−/−^ cells formed two separate clusters, establishing an NLRP3-dependent lipid signature (Figures 2A and 2B). The complete list of lipids and their abundance can be found in the Table S1.

Eicosanoids, including PG PGD_2_ and 11-hydroxyeicosatetraenoic acid (11-HETE), were among the species identified as more prevalent following NLRP3 activation (Figure 2C). We therefore quantified a variety of eicosanoids using their respective retention time and exact mass. Very similar patterns were observed regarding molecules such as PGF2α, PGE_2_, PGD_2_, 8isoPGF2α, 11-HETE, 15-HETE, 13-hydroxyoctadecadienoic acid (but not 13-hydroxydocosahexaenoate), thromboxane B2 (TxB_2_), and the eicosanoid precursor AA, with enhanced production in response to NLRP3 stimulation (Figures 2D–2M).

### AA inhibits the NLRP3 inflammasome

**Figure 3.**
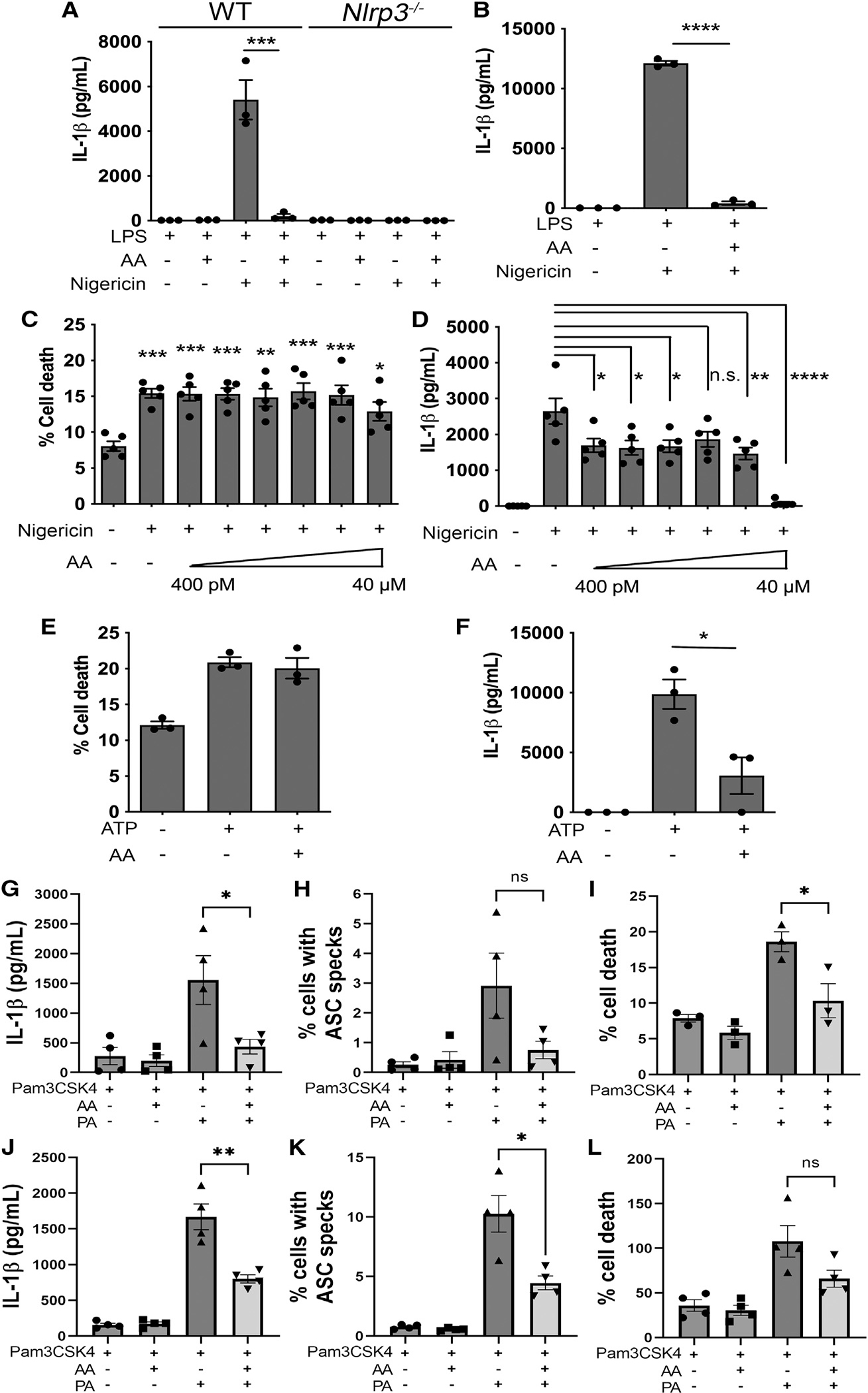
AA inhibits the NLRP3 inflammasome (A and B) IL-1β production in LPS-primed BMDMs after 1 h stimulation with 10 μM nigericin with or without 40 μM AA added after priming (A) or during priming (B). (C-F) Cellular viability (C and E) and IL-1β (D and F) produced by LPS-primed WT BMDMs after stimulation with nigericin (10 μM, 1 h) (C and D) or ATP (5 mM, 30 min) (E and F) in presence of 40 μM AA. (G-I) Cellular viability (G), ASC speck quantification (H), and IL-1β (I) produced by WT BMDMs primed with Pam3CSK4 (200 ng/mL, 4 h) after stimulation with palmitic acid (PA; 1 mM, 16 h) in presence of 40 μM AA. (J-L) Cellular viability (J), ASC speck quantification (K), and IL-1β (L) produced by THP-1 cells (differentiated with PMA, 200 ng/mL for 24 h followed by 24 h washout) primed with Pam3CSK4 (200 ng/mL, 4 h) after stimulation with PA (500 μM, 24 h) in presence of 40 μM AA. *p < 0.05, **p < 0.01, ***p < 0.001, and ****p < 0.0001 (one-way analysis of variance with Tukey’s multiple comparison test or Student’s unpaired t test). n.s., not significant. Data are from at least three independent experiments (mean and SEM).

We hypothesized that the inverse relationship between IL-1β and AA seen in the volunteer samples might be linked to an effect of AA, or its associated eicosanoid metabolites, inhibiting NLRP3 inflammasome activity. In our lipidomic analysis, stimulation of LPS-primed WT BMDMs with nigericin elicited a substantial increase in the production of AA release, in addition to eicosanoid production (Figure 2). We therefore compared the effects of AA with a number of eicosanoids on NLRP3 activation. Stimulation of LPS-primed BMDMs with nigericin in the presence of exogenous AA (40 μM) inhibited NLRP3-induced IL-1β production (Figure 3A). This inhibition was sustained when AA was present both during LPS priming and nigericin stimulation (Figure 3B). Titration analysis revealed that AA did not inhibit nigericin-stimulated, NLRP3-induced lytic cell death in BMDMs despite reducing IL-1β production even at low AA concentrations (Figures 3C and 3D). Similar results were found when NLRP3 was stimulated in BMDMs using ATP (Figures 3E and 3F).

Serum PA is elevated in metabolic syndrome.^23^ We therefore used this lipid to stimulate NLRP3 in the human THP-1 monocyte/macrophage cells differentiated with PMA to a macrophage-like phenotype. Cells were primed with TLR1/2 agonist PAM3CSK4 (200 ng/mL, 4 h), followed by NLRP3 stimulation with nigericin or PA.^24^ We measured IL-1β production but also immunolabeled the inflammasome adaptor protein ASC to determine the efficiency of ASC speck formation within these cells. NLRP3 activation recruits ASC prior to binding to, and activating, caspase-1.^5^ If AA inhibits NLRP3, then ASC speck formation, as well as IL-1β production, is expected to be reduced. AA inhibited IL-1β secretion and ASC speck formation but also lytic cell death in human THP-1 cells (Figures 3G–3I and S2). The inhibitory effect of AA was particularly noticeable when PA was used as the NLRP3 trigger, suggesting a potentially important role for AA in regulating lipid-driven inflammation (Figures 3J–3L). AA dose-dependently inhibited NLRP3-induced IL-1β production, ASC speck formation, and cell death after activation by PA in the concentration range of 10–40 μM (Figure S2).

**Figure 4.**
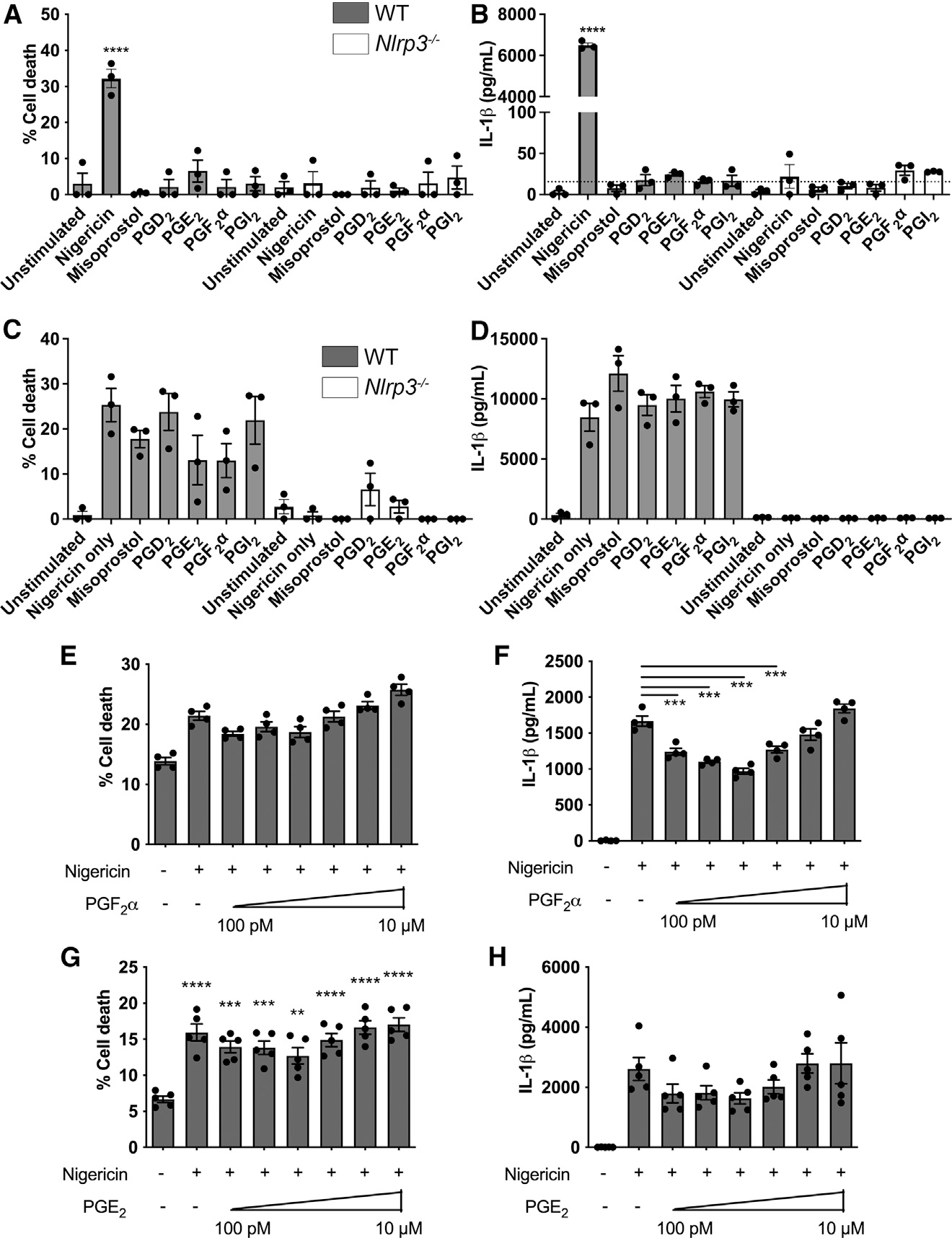
Individual eicosanoids have little effect on NLRP3 activity (A-D) Cell death (A and C), and IL-1β (B and D) produced by LPS-primed (200 ng/mL for 3 h) WT and *Nlrp3*^−/−^ BMDMs in response to 1 h stimulation with 1 μM misoprostol, PGD_2_, PGE_2_, PGF_2_α, and PGI_2_. (E-H) Cell death (E and G) and IL-1β (F and H) produced by LPS-primed WT BMDMs in response to 1 h stimulation with 10 mM nigericin in presence of increasing concentrations of PGF_2_α (E and F) and PGE_2_ (G and H). *p < 0.05, **p < 0.01, ***p < 0.001, and ****p < 0.0001 in comparison to untreated controls unless indicated otherwise (one-way analysis of variance with Tukey’s multiple comparison test). Dashed line represents the assay detection limit. Data are from three (A-D) or four (E-H) independent experiments (mean and SEM).

We then explored the possibility that the effect of AA on the NLRP3 inflammasome could be mediated by its metabolites. Specifically, we stimulated LPS-primed (3 h, 200 ng/mL) WT and *Nlrp3*^−/−^ BMDMs for 1 h with 1 μM freshly prepared eicosanoids/stable eicosanoid analogs (PGE_1_ analog misoprostol, PGD_2_, PGE_2_, PGF_2_α, and PGI_2_). Stimulation with these eicosanoids failed to induce cell death or IL-1β production (Figures 4A and 4B), suggesting that these individual eicosanoids do not induce inflammasome activation. The possibility remained, however, that individual eicosanoids could modify NLRP3 activity. To test this idea, LPS-primed (3 h, 200 ng/mL) WT and *Nlrp3*^−/−^ BMDMs were simultaneously stimulated with nigericin (10 μM, 1 h) and 1 μM freshly prepared eicosanoids. No statistically significant differences in cell death or IL-1β production were observed in comparison to nigericin-only controls (Figures 4C and 4D). Additional titration experiments were then performed in LPS-primed (3 h, 200 ng/mL) WT BMDMs stimulated with nigericin (10 μM for 1 h) in the presence of increasing concentrations of PGE_2_ or PGF_2_α (100 pM–10 μM). Again, no effect was seen on cell death (Figures 4E and 4G). Independent of concentration, PGE_2_ also did not significantly impact IL-1β production, although a trend toward lower IL-1β production at low PGE_2_ concentrations was observed, similar to previously reported data.^24^ Concentrations of PGF_2_α below 1 μM induced a significant decrease in IL-1β production (Figures 4F and 4H). Stimulation with increasing concentrations of PGE_2_ during LPS priming revealed an increase in IL-1β upon nigericin stimulation (in this experiment, PGE_2_ was present both during LPS priming and nigericin stimulation) (Figure S3). These results illustrated a complex regulatory network of individual eicosanoids upon inflammasome activation that is heavily influenced by experimental design and thus probably explains the apparent inconsistencies in the literature. It is possible that the net effect of the various COX-derived eicosanoids reflects a regulatory network on NLRP3 inflammasome activity consistent with an emerging role for lipids as modulators of the activity of this inflammasome. Collectively, however, our data uncover that AA itself is a potent inhibitor of the NLRP3 inflammasome.

### Inhibition of AA processing by COX selectively regulates canonical NLRP3 activity

**Figure 5.**
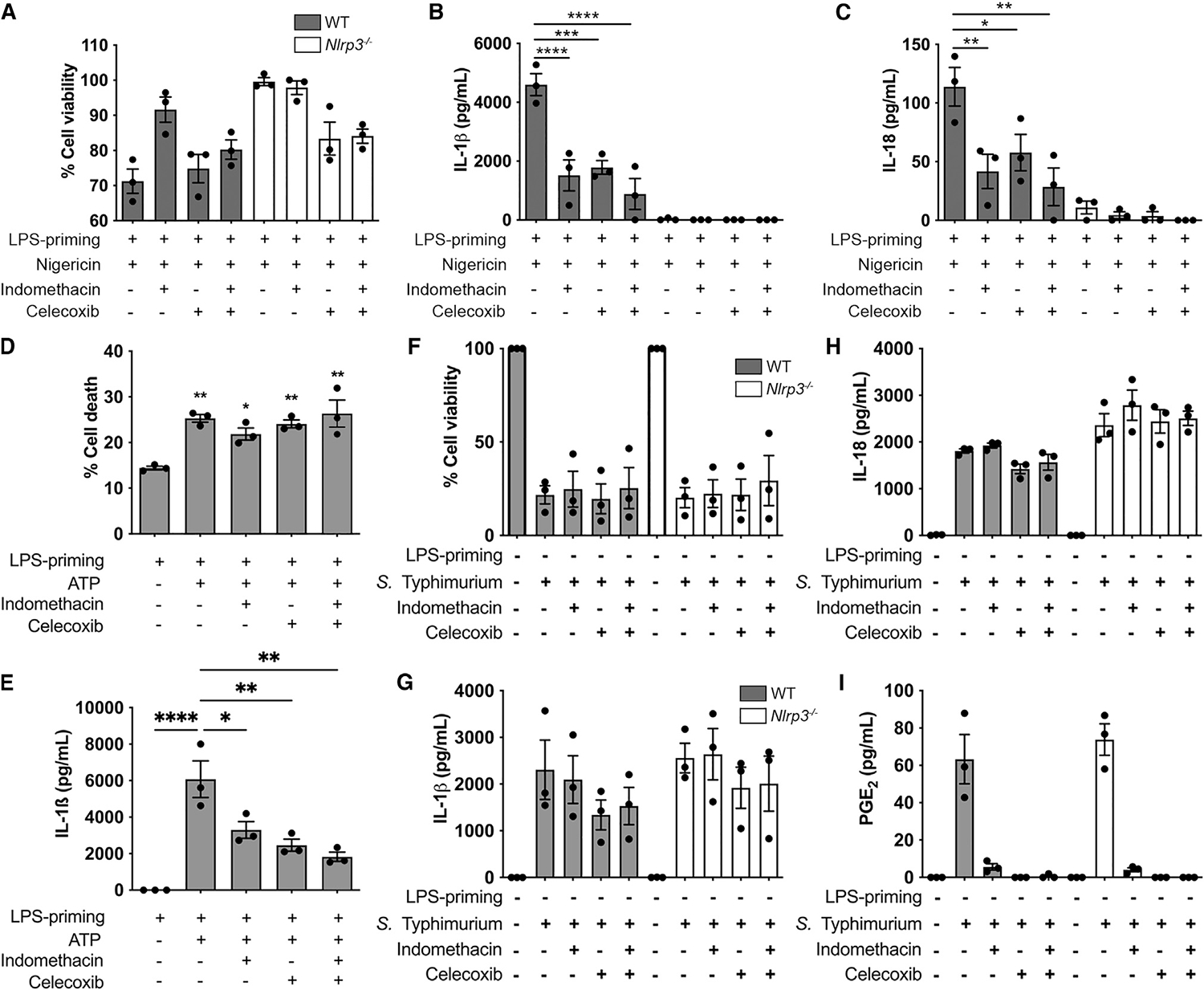
Cyclooxygenase (COX) activity regulates NLRP3 activity but not NLRC4 (A-H) Cellular viability (A and D), IL-1β (B and E), and IL-18 (C) produced by LPS-primed (200 ng/mL for 3 h) WT and *Nlrp3*^−/−^ BMDMs in response to NLRP3 stimulant nigericin (10 μM, 1 h) or ATP (5 mM, 30 min) in presence of COX inhibitors indometacin (100 μM) or celecoxib (10 μM). (F-I) Cellular viability (F), IL-1β (G), IL-18 (H), and PGE_2_ (I) produced by unprimed WT and *Nlrp3*^−/−^ BMDMs in response to *S*. Typhimurium infection (MOI 10, 2 h) in presence of COX inhibitors. *p < 0.05, **p < 0.01, ***p < 0.001, and ****p < 0.0001 (one-way analysis of variance with Tukey’s multiple comparison test). Data are from three independent experiments (mean and SEM).

AA is metabolized to eicosanoids, such as PGs, through the activity of COXs (COX-1 and COX-2). Inhibition of COX activity, by NSAIDs, suppresses eicosanoid production and enhances the availability of AA for processing through alternative metabolic pathways. We therefore investigated whether NSAID inhibition of AA metabolism through COX would also lead to NLRP3 inflammasome inhibition. Our lipidomic analysis showed that the COX-2 inhibitor celecoxib, while markedly reducing the production of eicosanoids such as PGF_2_aα, PGE_2_, PGD_2_, 11-HETE, 15-HETE, and TxB_2_ in response to nigericin stimulation, did not significantly suppress AA release (Figure S4). In previous work, COX inhibitors have been used to show that PGE_2_ stimulates IL-1β production via increasing *il1b* transcription^25,26^ or suppresses IL-1β production either due transcriptional or post-transcriptional effects.^16,24^ These inconsistencies could be due to differences in experimental design, as LPS priming of macrophages and COX inhibition were performed differently in each study, and COX metabolites can influence signaling pathways involved in pro-IL-1β expression.^27,28^ To circumvent this problem, we performed LPS stimulation and COX inhibition in conditions that induced similar levels of pro-IL-1β expression but with COX-2, NLRP3, and caspase-1 expressed at similar levels under all experimental conditions (Figures S5A–S5E). Using this system, LPS-primed WT and *Nlrp3*^−/−^ BMDMs were stimulated with the canonical NLRP3 stimulants nigericin or ATP in the presence or absence of COX-1/-2 inhibitors. COX inhibitors alone, in the absence of nigericin or ATP, did not induce inflammasome activity (Figures S5F–S5G). *Nlrp3*^−/−^ BMDMs, as expected, responded poorly to nigericin across all conditions, whereas WT BMDMs showed substantial IL-1β and IL-18 production upon treatment with either nigericin or ATP. In each case, we saw striking inhibition of both IL-1β and IL-18 production in the presence of COX inhibitors (Figures 5A–5E). The nonselective COX-1 and COX-2 inhibitor indomethacin and the COX-2 selective inhibitor celecoxib had similar effects on NLRP3 inflammasome activity. Collectively, our data support a model whereby COX inhibitors block NLRP3-induced inflammasome activity, most likely through the actions of AA.

NLRC4 stimulation triggers production of eicosanoids such as PGE2 both *in vivo* and in macrophages *in vitro.*^18,29^ To determine whether COX inhibition also regulates NLRC4 activation, we infected BMDMs with *Salmonella* Typhimurium (MOI 10) for 2 h in the presence of indomethacin or celecoxib. Under these conditions, *S*. Typhimurium activates primarily the NLRC4 inflammasome.^30^ During *Salmonella* infection of BMDMs, and similar to previous studies, COX-1/-2 inhibition blocked PGE2 production as expected but had no effect on NLRC4 inflammasome activity (Figures 5F–5I). These data suggest that COX inhibitors suppress NLRP3-induced inflammasome activity but do not impact the NLRC4 inflammasome.

### AA inhibits PLC activity and suppresses downstream protein kinases (PKs)

Our lipidomic analysis suggested that COX inhibition had little impact on AA abundance in macrophages, but both COX inhibition and AA addition during nigericin stimulation led to decreased IL-1β production. AA is released from the membrane predominantly by the activity of PLA_2_ but also by the action of PLC.^31^ NLRP3 is highly regulated by post-translational modifications (PTMs) including, for example, phosphorylation and ubiquitination.^7^ PLC activity can regulate NLRP3 PTM through JNK1-induced phosphorylation^7,^. We hypothesized that the presence of a “static” AA pool, for example in the presence of COX inhibitors, might trigger a negative feedback loop driven by classical product inhibition^32^ of PLC shutting off downstream PKs and hence NLRP3 activation.

**Figure 6.**
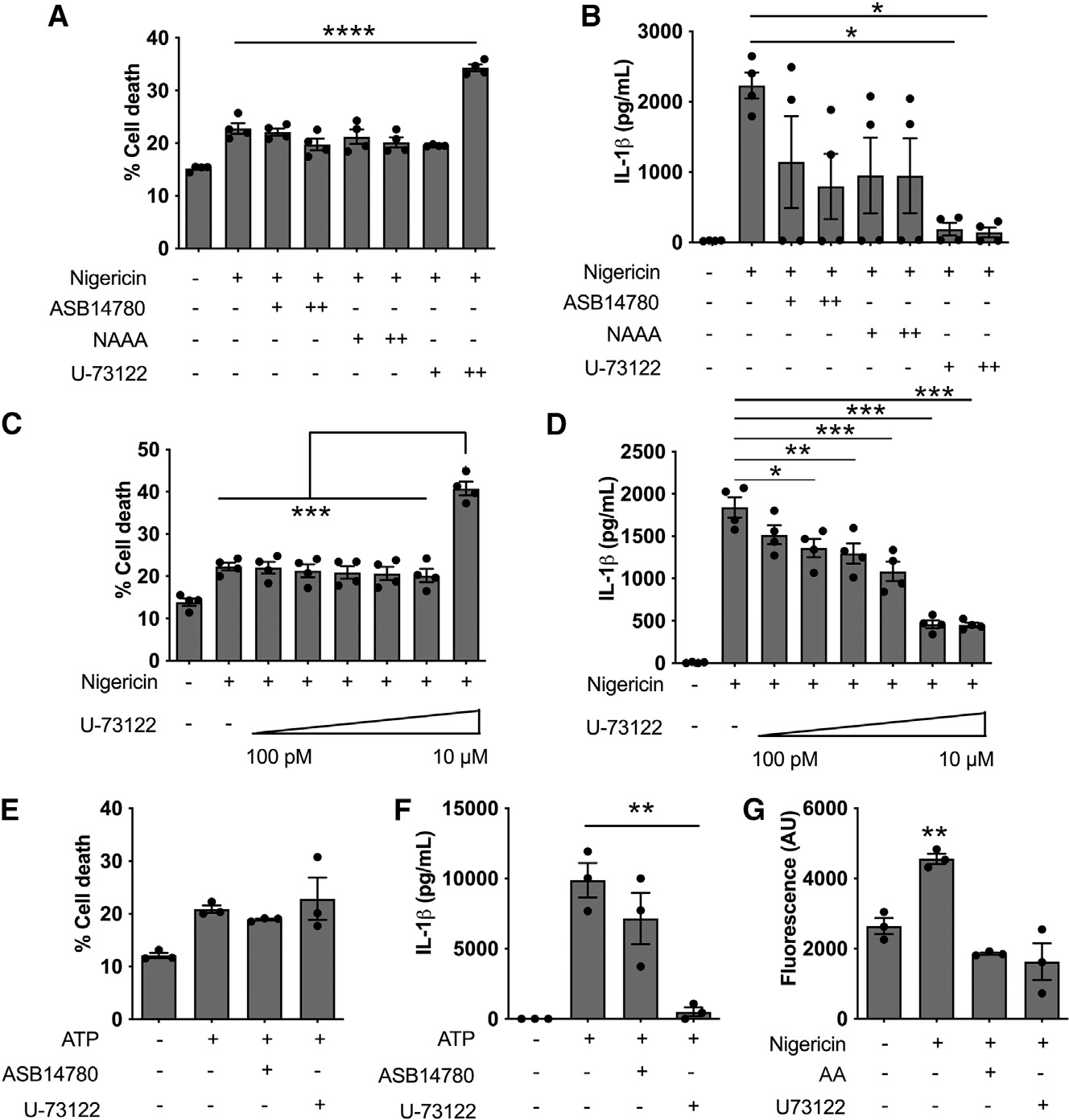
PLC is required for canonical NLRP3 activity (A and B) Cell death (A) and IL-1β production (B) in LPS-primed (200 ng/mL for 3 h) WT BMDMs in response to 1 h stimulation with 10 mM nigericin in presence of 1 (+) or 10 μM (++)PLA_2_ inhibitors ASB1414780 and NAAA or PLC inhibitor U-73122. (C and D) Cell death (C) and IL-1β (D) produced by LPS-primed WT BMDMs stimulated for 1 h with 10 mM nigericin in presence of increasing concentrations of PLC inhibitor U-73122. (E and F) Cell death (E) and IL-1β (F) produced by LPS-primed WT BMDMs stimulated for 30 min with 5 mM ATP in presence of 1 mM ASB14780 (PLA_2_ inhibitor) or 1 μM U-73122 (PLC inhibitor). (G) PLC activity of LPS-primed WT BMDMs stimulated with 10 μM nigericin for 1 h in presence of U-73122 or 40 μM AA. *p < 0.05, **p < 0.01, ***p < 0.001, and ****p < 0.0001 (one-way analysis of variance with Tukey’s multiple comparison test or Student’s unpaired t test). Data are from three (E–G) or four (A-D) independent experiments (mean and SEM).

To explore this idea, we investigated whether inhibition of PLA_2_ or PLC affects NLRP3-dependent IL-1β production and cell death. The PLA_2_ inhibitors ASB14780 and NAAA had no impact on NLRP3-induced IL-1β production, suggesting that PLA_2_ is not involved in regulating NLRP3 activation in response to nigericin (Figures 6A and 6B). Inhibition of PLC with U-73122, however, revealed a dose-dependent decrease in IL-1β production (Figure 6B). Cell death was increased at 10 μM inhibitor (Figure 6A), but this effect is due to toxicity unrelated to the inflammasome (Figure S6). Titration of the PLC inhibitor showed a dose-dependent decrease in nigericin-induced IL-1β production without affecting cell death (Figures 6C and 6D). Similarly, stimulation of the NLRP3 inflammasome with ATP was also regulated by PLC but not by PLA_2_ (Figures 6E and 6F).

Since product inhibition is a well-recognized negative feedback mechanism to control metabolic pathways,^32^ we next investigated whether AA can inhibit PLC activity. Some evidence for this to occur already exists,^33^ but whether a negative AA feedback loop operates in the context of inflammasome activation is unknown. To investigate this, we quantified PLC activity in protein extracts from LPS-primed WT BMDMs treated with nigericin (10 μM, 1 h) in the presence of AA or the PLC inhibitor (U-73122) as a control. Upon nigericin treatment, PLC activity increases significantly. Unsurprisingly, U-73122 suppressed PLC activity in response to nigericin, but AA also reduced nigericin-stimulated PLC activity (Figure 6G). These data suggested that activation of the canonical NLRP3 inflammasome is modified by PLC, but not PLA_2_, and that AA, by a product inhibition negative feedback loop, inhibits PLC activity.

**Figure 7.**
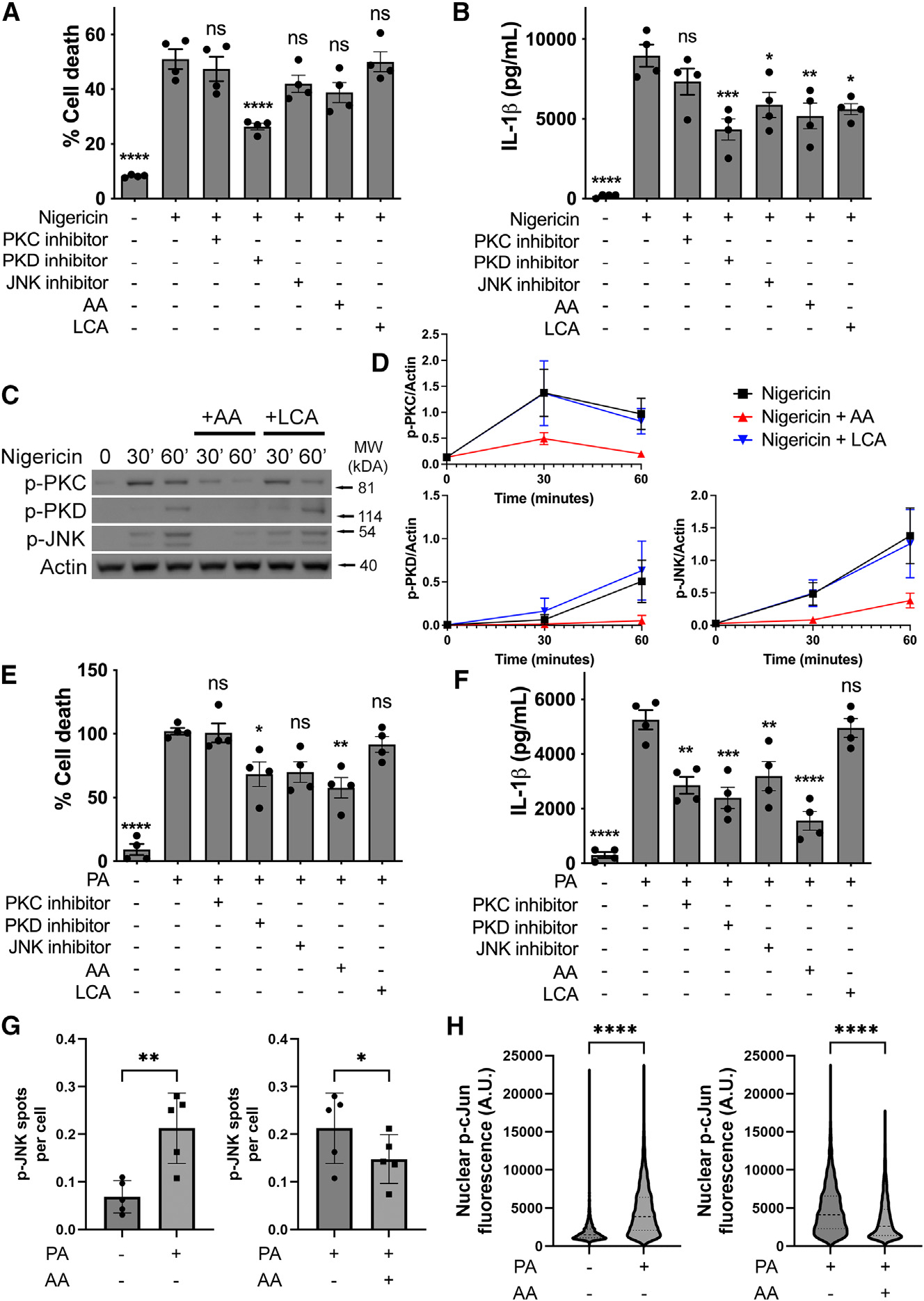
AA inhibits PKC, PKD, and JNK (A and B) Cell death (A) and IL-1β production (B) in THP-1 cells primed with LPS (200 ng/mL, 3 h) followed by stimulation with nigericin (10 μM, 1 h) in presence of PKC inhibitor (sotrastaurin, 10 μM), PKD inhibitor (CRT006601, 10 μM), JNK inhibitor (SP600125, 10 μM), AA (40 μM), or LCA (30 μM). (C and D) Immunoblots (C) and densitometric quantification (D) of THP-1 cells primed with LPS (200 ng/mL, 3 h) and stimulated with nigericin (10 μM) in presence of AA (40 μM) or LCA (30 μM) for up to 60 min. (E and F) Cell death (E) and IL-1β production (F) in THP-1 cells primed with Pam3CSK4 (200 ng/mL, 4 h) followed by stimulation with PA (500 μM, 16 h) in presence of PKC inhibitor (sotrastaurin, 10 μM), PKD inhibitor (CRT006601, 10 μM), JNK inhibitor (SP600125, 10 μM), AA (40 μM), or LCA (30 μM). (G and H) Quantification of *p*-JNK (G) and nuclear p-*c*-Jun (H) in THP-1 cells primed with Pam3CSK4 (200 ng/mL, 4 h) after stimulation with PA (500 μM, 24 h) in presence of 40 μM AA. THP-1 cells were differentiated with PMA (200 ng/mL) for 24 h followed by 24 h washout prior to the experiments. *p < 0.05, **p < 0.01, ***p < 0.001, and ****p < 0.0001 in comparison to nigericin-treated cells (A and B) or PA-treated cells (E and F) unless indicated otherwise (one-way analysis of variance with Tukey’s multiple comparison test). n.s., not significant. Data are from three (C and D), four (A, B, E, and F), or five (G and H) independent experiments (mean and SEM).

PLC stimulates the activation of PKs such as PKC, PKD, and JNK1,^31,34,35^ which, in turn, upregulates NLRP3 activation.^8,10,36,37^ We therefore tested whether AA could suppress NLRP3-induced JNK phosphorylation. In THP-1 cells stimulated with nigericin, inhibition of either PKD or JNK led to decreased NLRP3 activation (Figures 7A and 7B). Accordingly, the presence of AA inhibited PKC, PKD, and JNK activation, as suggested by decreased phosphorylation (Figures 7C and 7D). The bile salt lithocholic acid (LCA) also inhibits NLRP3 inflammasome activation.^38^ LCA, however, does not affect PKC, PKD, and JNK phosphorylation, suggesting that AA and bile salts inhibit the NLRP3 inflammasome by distinct mechanisms (Figures 7A–7D).

Similarly, in PA-stimulated THP-1 cells, inhibition of PKC, PKD, and JNK led to impaired NLRP3 activation (Figures 7E and 7F). In response to PA, JNK was phosphorylated and activated as demonstrated by phosphorylation of the JNK target c-Jun. AA suppressed both PA-induced JNK phosphorylation and c-Jun phosphorylation (Figures 7G and 7H). These data identified that AA drives a negative feedback loop, via product inhibition, that suppresses NLRP3 activation through JNK phosphorylation. This provides a mechanistic basis explaining how NLRP3 activity may be regulated by AA either in response to NSAID (COX) inhibition or fasting.

## DISCUSSION

Here, we show that AA inhibits the NLRP3 inflammasome, through a PLC-JNK-dependent mechanism, to supress IL-1β production under physiological conditions. This provides a mechanistic basis for how dietary manipulation such as fasting influences the inflammatory state and is likely to be critical for reducing the metaflammation underpinning many diseases induced by the WD. Fasting can differentially regulate NLRP3 activity,^2^ and elevated IL-1β is observed after feeding,^39,40^ but the mechanism for this is unclear. It is increasingly clear that lipids are important regulators of NLRP3 activity,^12,13^ but how this occurs is still poorly understood. Here, we show that in a fasting subject cohort where IL-1β is suppressed, AA is elevated, and this effect is reversed upon feeding. While we do not have direct *in vivo* evidence that AA suppresses IL-1β production, our data highlighted the possibility that AA, or one of its metabolites, might be an NLRP3 regulator. Unexpectedly, we showed *in vitro* that AA itself inhibits activation of the NLRP3 inflammasome, while AA metabolites do not. COX inhibitors, which decrease the production of AA eicosanoid metabolites, but not AA itself, also decrease NLRP3 activity, providing further evidence that the NLRP3-inhibitory effect is a direct result of AA rather than a metabolite.

We hypothesized that AA-driven NLRP3 inhibition might occur by product inhibition of the enzymes that liberate AA from the plasma membrane, which are PLA_2_- and, to a lesser extent, PLC dependent. Our data show that AA inhibits PLC activity, supporting our hypothesis that a product inhibition negative feedback loop occurs.^33^ Links between PLC and canonical inflammasome activity were reported,^41,42^ but how precisely PLC activates the NLRP3 inflammasome remains to be fully elucidated. Here, we hypothesized that PLC regulates NLRP3 activity via PKC, PKD, and JNK. PLC drives the generation of the second messenger diacylglycerol, which activates PKC and PKD.^31,34,35^ The PLC/PKC axis activates JNK1, which we, and others, have shown is important in activating NLRP3.^8,10^ Additionally, PKD directly phosphorylates NLRP3, enhancing its activity.^37^ Our data supported this hypothesis, as PLC inhibitors, as well as AA, inhibit JNK1 activation, evidence that the PLC-JNK axis is an important regulator of NLRP3 activity.

We also observed that while AA inhibited NLRP3-mediated IL-1β production, its effect on cell death was marginal. Recently published work has shown that dendritic cells release IL-1β in the absence of cell death upon stimulation with oxidized phospholipids (OxPaPC).^43^ The mechanisms underpinning this are due to GSDMD-perforated plasma membranes being repaired by triggering the assembly of the endosomal sorting complexes required for transport III (ESCRT III) machinery at the plasma membrane, where it removes the GSDMD pores by shedding them into vesicles, thus preventing GSDMD-mediated pyroptosis.^44^ We speculate that a similar GSDMD pore repair process may explain our observations, although the relationships between AA, ESCRT III, and NLRP3 remain to be elucidated.

Our work demonstrates a mechanism by which the NSAID COX inhibitors have broad anti-inflammatory effects: through AA inhibition of NLRP3 activity. A number of papers have investigated how COX-derived eicosanoids might interface with inflammasomes. Eicosanoid regulation of IL-1β has, for the most part, focused on transcriptional events.^16,25,26^ Here, COX inhibition decreased NLRP3-mediated IL-1β and IL-18 production. Our analysis indicated that individual eicosanoids had some minor regulatory effects on canonical NLRP3 activation. Eicosanoids such as PGE_2_ and PGF_2α_ showed some decrease in the production of IL-1β in LPS-primed BMDMs stimulated with nigericin similar to data from a previous report where PGE_2_ inhibits the inflammasome via PKA and phosphorylation of NLRP3.^23^ Others report that PGE_2_ stimulates IL-1β production in response to *Tytius serralatus* venom via PKA activation.^45^ Our data show that COX inhibition decreases the production of PGE_2_ (and other eicosanoids) but not AA, suggesting that this lipid, rather than an eicosanoid, is regulating NLRP3 activation. The apparent discrepancies in the data around eicosanoid regulation of NLRP3 inflammasome activity suggest there is a complex lipid regulatory network that may have a concentration dependency.

Our data also support an important metabolic loop whereby fasting elevates AA, which feeds back on to the inflammasome, suppressing IL-1β. Elegant data suggest that in adults, IL-1β production is produced as a physiological response to food.^3^ Our data here show that AA is enhanced in adults upon fasting, when IL-1β is suppressed. This suggests that rather than IL-1β always being involved in pathological responses, in response to feeding, there is a novel, physiological regulatory loop between IL-1β, NLRP3, and AA. Since we have shown that NLRP3 inflammasome activation yields an increase in AA, this regulatory loop may prevent excessive inflammasome activation under normal physiologic conditions. We hypothesize that this differs in the context of the WD, where the NLRP3 inflammasome is chronically activated, and hence IL-1β is contributing to the pathology of long-term inflammation. How AA is regulated in these people is unclear, but it is tempting to speculate that the success of intermittent fasting diets may involve fasting-triggered AA production to suppress inflammasome activity, thereby reducing metaflammation associated with WD metabolic syndrome.

In conclusion, we provide data to suggest that AA is an important physiological inhibitor of the NLRP3 inflammasome. The elevation of AA in plasma lipids from fasting volunteers provides a mechanism to explain the drop in IL-1β production from PBMCs from these people and, potentially, one way in which fasting has beneficial anti-inflammatory effects. Our data also suggest a mechanism by which NSAIDs are anti-inflammatory and identify that AA may have a previously unappreciated role as a primary signaling lipid.

### Limitations of the study

Our study demonstrates a mechanism for AA-mediated inflammasome inhibition *in vitro*. While our *in vivo* data from human volunteers observed elevated AA and reduced IL-1β, in agreement with our *in vitro* data, this evidence is indirect. Further studies are required to unambiguously link AA to reduced inflammation *in vivo* and validate the mechanisms we proposed in this article.

## STAR★METHODS

### RESOURCE AVAILABILITY

#### Lead contact

Further information and resource requests can be directed to and will be fulfilled by the lead contact Clare E. Bryant (ceb27@cam.ac.uk).

#### Materials availability

Mouse lines and cell lines used in this study are available from the lead contact with a completed Materials Transfer Agreement.

#### Data and code availability

- The uncropped immunoblots, quantitative and lipidomic data have been deposited at Mendeley Data and are publicly available as of the date of publication. DOIs are listed in the Key resources table.
- This paper does not report original code.
- Any additional information required to reanalyze the data reported in this paper is available from the lead contact upon request.

### EXPERIMENTAL MODEL AND STUDY PARTICIPANT DETAILS

#### Mice

WT C57BL/6 mice were obtained from Charles River, UK. *Nlrp3*^*−/−*^ mice on a C57BL/6 background were produced by Millenium Pharmaceuticals and obtained from Kate Fitzgerald (University of Massachusetts). All animals were housed in a pathogen-free facility and all work involving live animals complied with the University of Cambridge Ethics Committee regulations under Home Office Project License number 80/2572. This study used male and female mice in similar proportions, all ranging from 8 to 12-weeks old.

#### Human studies

The fasting and refeeding clinical study in healthy volunteers was approved by the US National Institutes of Health Intramural Institutional Review Board (ClinicalTrials Identifier No. NCT02719899).^47^ In the first visit, volunteers were screened in the ambulatory clinic and signed informed consent for the protocol before enrolling in the study. In a second visit, blood was drawn from overnight-fasted participants to establish the baseline immune response. Next, overnight-fasted participants consumed a 500-calorie meal before 8 a.m. and fasted for 24h with unrestricted water intake. After 24h, blood was draw (fasted samples), the participants ate a 500-calorie meal and after 3h, blood was draw once again (refed samples).^47^ The PBMCs were extracted following the 24h fast and 3h after refeeding and incubated with ATP (3 mM) for 30 min, prior to the measurement of IL-1β release by ELISA assays.^2^ Serum from these same samples were employed to measure circulating arachidonic acid levels.

### METHOD DETAILS

#### Lipid extraction and mass spectrometry

Lipid extraction and mass spectrometry extractions were performed as follows^18^: 2.5 mL of isopropanol/hexane/acetic acid solution (20:30:2, v/v/v) at 4°C and 10 ng of COX and LOX LC/MS mixture (CAY19228–1, Cayman Chemical) were added to 1 mL supernatants from stimulated or stimulated BMDMs and kept on ice for 10 min. Samples were then vortexed for 15 s, 2.5 mL of hexane (4°C) was added and the solution vortexed for another 15 s. The samples were subjected to centrifugation (5 min, 4°C, 900 G), the organic layer collected. The aqueous fraction was re-extracted by addition of 3.75 mL chloroform/methanol (1:2, v/v), vortexed for 15 s and 1.25 mL of chloroform was added. Samples were then vortexed for 15 s, 1.25 mL of water was added, vortexed again, and centrifuged (5 min, 4°C, 900 G). The organic layer was combined with the organic layer from the first extraction, dried under nitrogen, and analyzed by liquid chromatography/drift tube ion mobility coupled with high resolution mass spectrometry (LC/DTIM-MS) (Agilent 6560 IM QTOF MS (Agilent Technologies) with a reverse-phase ACQUITY CSH C18 column (Waters Company). Annotation and identification of lipids was performed with an adaption of the KniMet pipeline^22^ with combined annotation comprising the LIPID MAPS Structure Database and an internal lipid library.^19^

#### Cell isolation and culture

C57BL/6 mice were killed by cervical dislocation, ethanol 70% was sprayed for sterilization and the skin around the leg was removed. Afterward, the leg was removed and placed in DMEM (Dulbecco’s modified Eagle’s medium) on ice. In a laminar flow cabinet, the muscle was removed, the tibia and femur were separated at the knee ligament, and the proximal and distal epiphysis removed. For bone marrow derived macrophages (BMDM) culture, bone marrow was flushed out using BMDM growth media (DMEM supplemented with 10% HyClone (Thermo Fisher Scientific), 20% L929 conditioned media and 5 mM L-Glutamine (Sigma)), and the collected cells were centrifuged at 300 × G for 10 min at 15°C and resuspended in BMDM growth media. The cells were then cultured at 37°C under 5% CO_2_ atmospheric conditions, with the addition of equal volume of the appropriate media after 2 days in culture and completely replenishing the media after 4 days in culture. Every experiment was conducted using cells with 7–9 days in culture.

L929 conditioned media was prepared by growing L929 cells to confluence for 2 weeks, in RPMI 1640 (Sigma) supplemented with 10% Hyclone and 5 mM L-Glutamine. The culture supernatant was collected and sterilized by filtration in 0.22 μm filters (Milipore).

#### Cell stimulation and infection

*Salmonella* Typhimurium strain SL1344^46^ were cultivated to log phase by pre-culturing the bacteria for 17.5 h in 5 mL LB broth (Sigma) at 37°C and 200 rpm, followed by a 1 in 10 dilution of the pre-culture in LB broth and further culture for 2 h. The bacteria were then centrifuged for 10 min at 4.300 × G and washed in BMDM growth media and allowed to infect cells at the indicated multiplicity of infection (MOI) for an hour at 37°C and 5% CO_2_. For the 2 and 6 h timepoints, the infection was followed by washing and incubation with media containing 50 μg/mL gentamicin (Sigma) for another hour. For the 6 h timepoint, media was replaced by supplemented DMEM containing 10 μg/mL gentamicin and incubated at 37°C for another 4 h. Culture supernatants were collected for every timepoint for cytokine quantitation.

Selected experiments required priming with LPS or Pam3CSK4. This was performed by incubation of cells in growth media containing 200 ng/mL ultrapure LPS from *Escherichia coli* O111:B4 (Invivogen) for 3 h or 200 ng/mL Pam3CSK4 (Invivogen) for 4 h at 37°C and 5% CO_2_, followed by successive washing in media alone. Before priming, COX inhibition experiments included a pre-incubation step of 30 min with indomethacin 100 mM (Sigma) or celecoxib 10 μM (Sigma). COX inhibitors were also present at these concentrations during LPS priming and stimulations.

For stimulation experiments, the LPS-primed cells were incubated with Nigericin 10 μM (Sigma) for 1 h or ATP 5 mM (Sigma) for 30 min. In selected assays, nigericin 10 μM and prostaglandins (obtained from Tocris) at 1 μM (unless otherwise stated) were added simultaneously and incubated for 1 h. A pre-incubation step of 30 min was performed after priming in experiments containing arachidonic acid 40 μM (Tocris), U-73122 1 μM (Sigma), ASB14780 1 μM (Sigma), N-(*p*-Amylcinnamoyl)anthranilic acid 1 μM (Sigma), sotrastaurin 10 μM (abcam), CRT0066101 10 μM (Tocris), SP600125 10 μM (Tocris), LCA 30 μM (Sigma).

#### Cellular viability assays

BMDM cytotoxicity was measure using the CytoTox 96 Non-Radioactive Cytotoxicity Assay (Promega). Briefly, after cellular infection and stimulation as indicated above, the adhered cells were washed three times in non-supplemented DMEM, incubated with 40 μL per well of Triton X-100 0.5% for 15 min at 4°C. The cells were then scrapped from the wells and 10 μL of each well was transferred to another plate containing 105 μL Triton X-100 1.2% per well and incubated at 37°C for an hour, diluted in PBS if necessary and the CytoTox reagent was used as described by the manufacturer. Cellular viability was then calculated in relation to the uninfected control containing 200.000 cells (100% viability). To quantify cell death, the supernatants of stimulated cells were collected and LDH activity was measured using the CytoTox 96 Non-Radioactive Cytotoxicity Assay (Promega), in relation to a 100% cell lysis control done by lysing 200.000 cells with the lysis reagent present in the CytoTox kit.

#### Cytokine quantification

Secreted cytokines were quantified by enzyme linked immunosorbent assay (ELISA) using the experiments supernatants after appropriate dilution in growth media. All cytokines were measured according to the manufacturer’s instructions. For IL-1β the kit OptEIA Mouse IL-1β Set (BD Biosciences) and Human IL-1β DuoSet (R&D Systems) were used. For IL-18 the kit mouse IL-18 ELISA (MBL International) was used.

#### Immunoblots

After stimulation, cells were lysed for 10 min in ice using buffer containing 10 mM Tris pH 7.4, 150 mM NaCl, 5 mM EDTA, 1% Triton X-100, 10 mM NaF, 1 mM NaVO_4_, 20 mM PMSF, Phosphatase inhibitor cocktail 3 (1 in 100 dilution, Sigma) and Protease inhibitor cocktail (1 in 100 dilution, P8340, Sigma). Protein levels were quantified using Pierce BCA Protein Assay Kit (Life Technologies) and adjusted to 500 μg/mL for immunoblotting. The samples were then incubated for 5 min at 100°C with Pierce Lane Marker Reducing Sample Buffer (Life Technologies). Gels were loaded with 20 μL of the sample per lane, with a final protein mass of 10 μg.

Immunoblots were probed using the following primary antibodies: caspase-1 p10 (mouse) (sc-514, Santa Cruz) 1 in 500; IL-1β (goat) (AF-401, R&D Systems) 1 in 1000; β-Actin (mouse) (AB3280, ABCAM) 1 in 2500; NLRP3 (rat) (MAB7578-SP, R&D Systems) 1 in 2000; COX2 (goat) (AF4198, R&D Systems). The secondary antibodies used were: anti-goat IgG-HRP (sc-2922, Santa Cruz) 1 in 5000; anti-mouse IgG-HRP (7076, Cell Signaling) 1 in 6000; anti-rabbit IgG-HRP (A24537, Thermo Scientific) 1 in 6000 as appropriate; anti-rat IgG-HRP (7077S, Cell Signaling) 1 in 5000.

#### PLC activity assay

10.000.000 BMDMs were plated in petri dishes overnight and primed with LPS as described above. The cells were then pre-incubated with arachidonic acid or U-73122 as described above and next stimulated with nigericin 10 μM for 1 h with or without inhibitors. The cells were then collected and lysed for 10 min in ice using buffer containing 10 mM Tris pH 7.4, 150 mM NaCl, 5 mM EDTA, 1% Triton X-100, 10 mM NaF, 1 mM NaVO_4_, 20 mM PMSF, Phosphatase inhibitor cocktail 3 (1 in 100 dilution, Sigma) and Protease inhibitor cocktail (1 in 100 dilution, P8340, Sigma). PLC activity was then measured with EnzChek Direct Phospholipase C Assay Kit (Thermo) as indicated by the manufacturer.

### QUANTIFICATION AND STATISTICAL ANALYSIS

Data analysis was done using the software Prism 6.0 (GraphPad Software) as indicated in each individual experiment. In summary, statistical difference between two groups was determined using unpaired t test, differences between multiple groups were determined using one-way analysis of variance (ANOVA) with Tukey’s post-test. In this work, a p value below 0.05 was considered significant. Analysis of metabolomic data was done using SIMCA 15 (Sartorius).

## Supplementary Material

1

2

## Figures and Tables

**KEY RESOURCES TABLE:** 

REAGENT or RESOURCE	SOURCE	IDENTIFIER

Antibodies		

Rabbit pAb anti-Caspase-1 p10	Santa Cruz	sc-514; RRID AB_2068895
Goat pAb anti-IL-1 beta/IL-1F2	R&D	AF-401; RRID AB_416684
Mouse mAb anti-Actin	Abcam	AB3280; RRID AB_303668
Rat mAb anti-NLRP3	R&D	MAB7578; RRID AB_2889405
Goat pAb anti-COX2	R&D	AF4198; RRID AB_2229909
Rabbit pAb anti-Goat IgG Peroxidase	Santa Cruz	sc-2922; RRID AB_656965
Horse pAb anti-mouse IgG Peroxidase	Cell Signaling	7076; RRID AB_330924
Goat pAb anti-Rabbit IgG (whole molecule) Peroxidase	Sigma-Aldrich	7077; RRID AB_10694715
Rabbit pAb anti-phospho-PKC (pan)	Cell Signaling	9371; RRID AB_2168219
Rabbit pAb anti-phospho-PKD	Cell Signaling	2051; RRID AB_330841
Rabbit pAb anti-phospho-JNK	Cell Signaling	4668; RRID AB_823588

Bacterial and virus strains		

*Salmonella enterica* Typhimurium SL1344	Hoiseth and Stocker^46^	N/A

Biological samples		

Human blood samples	From subject cohort – ClinicalTrials.gov ID NCT02719899	N/A

Chemicals, peptides, and recombinant proteins		

Acetic Acid	Millipore Sigma	5330010050
Arachidonic Acid	Tocris	2756
Arachidonic Acid	Sigma-Aldrich	10931
ASB-14780	Sigma-Aldrich	SML1913
ATP	Sigma-Aldrich	A1852
Celecoxib	Millipore Sigma	SML3031
Chloroform	Millipore Sigma	288306
CRT0066101	Tocris	4975
Deutered COX and LOX LC-MS Mixture	Cayman Chemical	19228
Dulbecco’s Modification of Eagle’s Medium	Corning	10–013-CV
Dulbecco’s Phosphate-Buffered Saline	Corning	21–031-CV
Gentamicin	Millipore Sigma	G1397
Halt Phosphatase Inhibitor Cocktail (100x)	Thermo-Fisher	1862495
Halt Protease Inhibitor Cocktail (100x)	Thermo-Fisher	87786
Hexane	Millipore Sigma	1043911000
HyClone Fetal Bovine Serum	Fisher Scientific	SH3008803
Indomethacin	Millipore Sigma	I7378
Isopropanol	Millipore Sigma	1027811000
L-Glutamine	Millipore Sigma	G7513
LB Broth (Miller)	Sigma-Aldrich	L3522
LB Broth with Agar (Miller)	Sigma-Aldrich	L3147
Lithocholic Acid	Millipore Sigma	L6250
Methanol	Millipore Sigma	1.06035
Misoprostol	Tocris	2297
N-(*p*-Amylcinnamoyl)anthranilic acid	Sigma-Aldrich	A8486
Nigericin	Sigma-Aldrich	N7143
NP-40 (Nonidet P-40 Substitute)	Boston Bioproducts	#P-877
Palmitic Acid	Millipore Sigma	P0500
Pam3CSK4	Invivogen	tlrl-pms
Penicilin Streptomycin Solution, 100x	Corning	30–002-CI
Pierce^™^ Lane Marker Reducing Sample Buffer	Thermo-Fisher	39000
Prostaglandin D2	Cayman Chemical	12010
Prostaglandin E2	Tocris	2296
Prostaglandin F2alpha	Tocris	4214
Prostaglandin I2	Tocris	2989
Sotrastaurim	Abcam	ab219867
SP600125	Tocris	1496
TMB Substrate Reagent Set	BD	555214
Ultrapure LPS, *E. coli* O111:B4	Invivogen	tlrl-3pelps
U-73122	Sigma-Aldrich	662035

Critical commercial assays		

CytoTox 96^(R)^ Non-Radioactive Cytotoxicity Assay	Promega	G1780
Clarity Max Western ECL Substrate	Bio-Rad	1705062
EnzChek^(TM)^ Direct Phospholipase C Assay Kit	Invitrogen	E10215
Human IL-1Beta/IL-1F2 DuoSet ELISA	R&D Systems	DY201
Mouse IL-1Beta/IL-1F2 DuoSet ELISA	R&D Systems	DY401
Mouse IL-18 Elisa kit	MBL International	7625
Pierce BCA Protein Assay Kit	Thermo Scientific	23225
PGE2 ELISA kit	Enzo Life Sciences	ADI-900–001

Deposited data		

Raw data	This paper	Mendeley Data: https://doi.org/10.17632/vtpjtgm2tw.1

Experimental models: Cell lines		

THP-1	ATCC	ATCC TIB-202

Experimental models: Organisms/strains		
Mouse: C57BL/6	Charles Rivers	N/A
Mouse: C57BL/6 NLRP3 Knockout	Millenium Pharmaceuticals	N/A

Software and algorithms		

GraphPad Prism	Graphpad Software	www.graphpad.com
ImageJ	NIH	imagej.nih.gov/ij/
SIMCA	Sartorius	www.sartorius.com

## Highlights

- In fasting compared to fed subjects, plasma IL-1β is lower and arachidonic acid (AA) is higher
- Exogenous AA impairs NLRP3 inflammasome activity in human and mouse macrophages
- AA inhibits phospholipase C and reduces JNK stimulation and hence NLRP3 activity
