# Supplementary material for: Arachidonic acid inhibition of the NLRP3 inflammasome is a mechanism to explain the anti-inflammatory effects of fasting
Source: Cell Rep. Author manuscript; Available in PMC 2024 Mar 15. (PMC10940735; doi:10.1016/j.celrep.2024.113700)
Supplement: 1 [file NIHMS1970867-supplement-1.pdf]

Cell Reports, Volume *43*

Supplemental information

Arachidonic acid inhibition of the NLRP3 inflammasome is a mechanism to explain the anti-inflammatory effects of fasting

Milton Pereira, Jonathan Liang, Joy Edwards-Hicks, Allison M. Meadows, Christine Hinz, Sonia Liggi, Matthias Hepprich, Jonathan M. Mudry, Kim Han, Julian L. Griffin, Iain Fraser, Michael N. Sack, Christoph Hess, and Clare E. Bryant

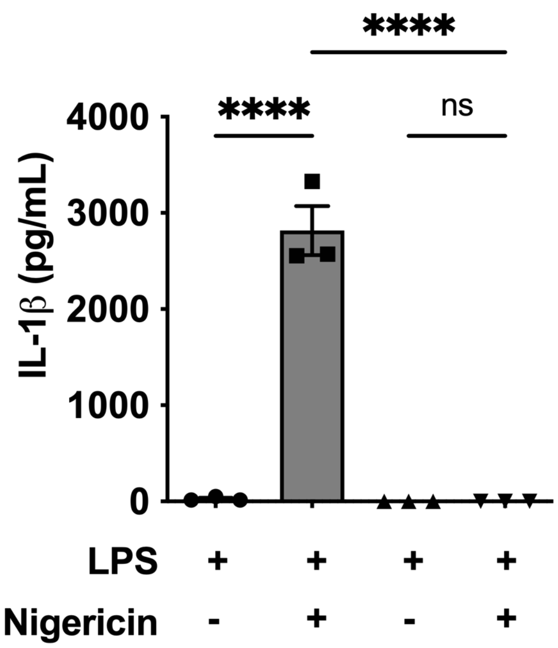

Figure S1: IL-1β production in nigericin-treated WT and *Nlrp3*$^{-/-}$ BMDMs, related to Figure 2. IL-1β present in culture supernatants of LPS-primed (200 ng/mL, 3 hours) WT and *Nlrp3*$^{-/-}$ BMDMs stimulated with nigericin (10 µM, one hour). Data are representative of three independent experiments, **** p < 0.0001 (one-way analysis of variance with Tukey's multiple comparison test). Data from three independent experiments (mean and s.e.m.).

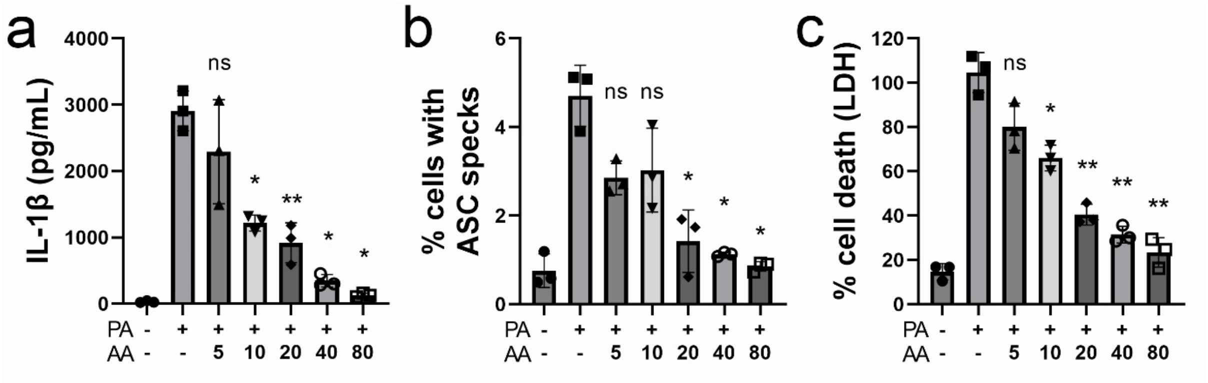

Figure S2: Dose-dependent inhibition of NLRP3 inflammasome activity by arachidonic acid, related to Fig. 3. THP-1 cells were differentiated with PMA (200 ng/mL) for 24 hours, rested for 24 hours, then primed with Pam3CSK4 (200 ng/mL) for 4 hours. Cells were then treated with AA at the indicated concentrations (µM) for 30 minutes prior to addition of PA (500 µM, BSA-conjugated). After 24 hours, supernatant was collected and cells were fixed and immunostained for ASC. (a) IL-1β in supernatant, (b) cells with ASC specks, and (c) cell death by LDH release assay were measured. Data are representative of three independent experiments, * p < 0.05, ** p < 0.01 (one-way analysis of variance with Tukey's multiple comparison test). n.s., not significant.

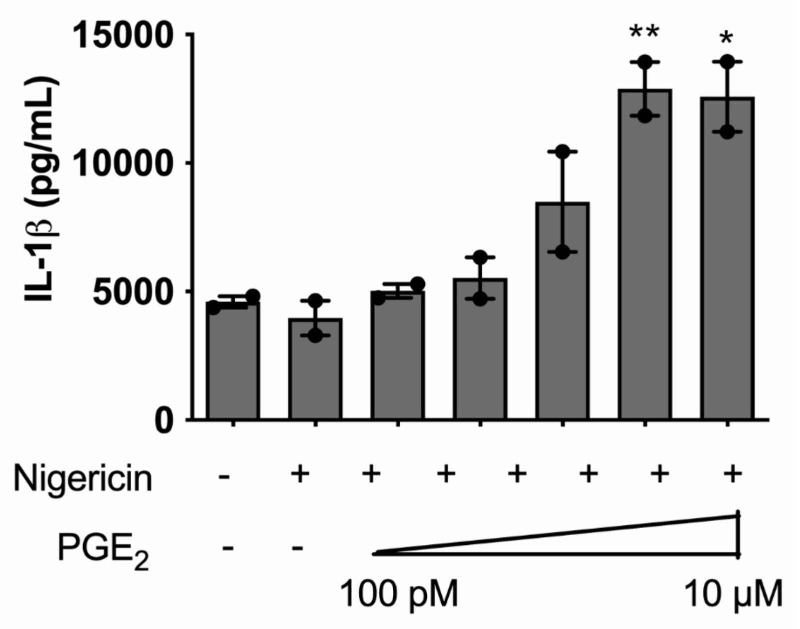

Figure S3: Effects of PGE_2 on NLRP3 activity, related to Fig. 4. Increasing concentrations of PGE_2 were added during LPS-priming (200 ng/mL for 3 hours) of WT BMDMs and then stimulated with 10 μM nigericin in presence of PGE_2 and IL-1β in the supernatant quantified. * $p < 0.05$, ** $p < 0.01$ (one-way analysis of variance with Tukey's multiple comparison test). Data from two independent experiments (mean and s.e.m.).

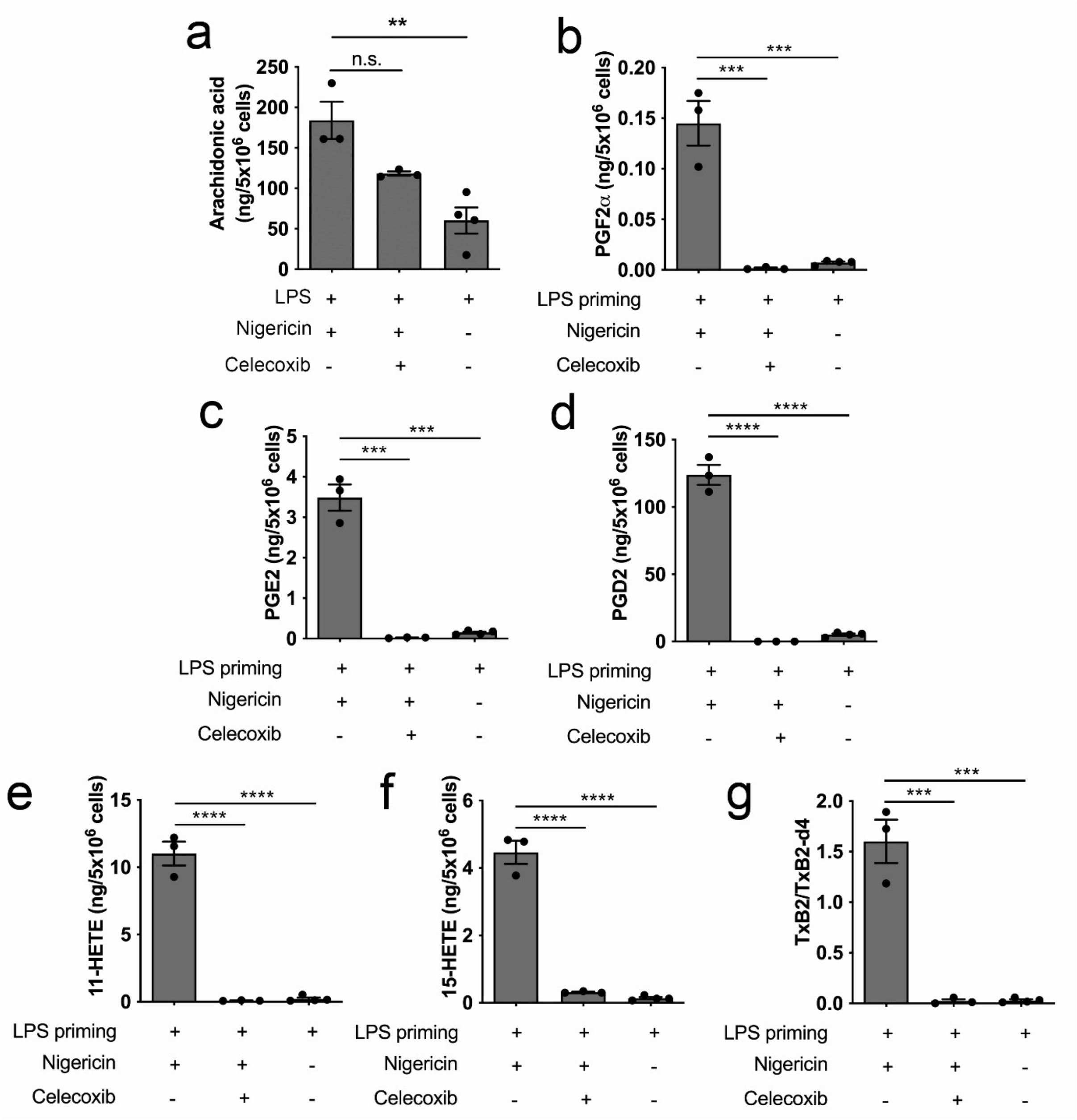

Figure S4: Celecoxib abolishes the production of COX-derived eicosanoids. (a-g) LC/DTIM-MS quantification of arachidonic acid (a), PGF2α (b), PGE2 (c), PGD2 (d), 11-HETE (e), 15-HETE (f), and TxB2 (g) after one-hour treatment of LPS-primed (200 ng mL^{-1} for 3 hours) WT BMDMs with 10 µM nigericin with or without 10 µM celecoxib. *** $p < 0.001$, **** $p < 0.0001$ (one-way analysis of variance with Tukey's multiple comparison test). Data from three independent experiments (mean and s.e.m.).

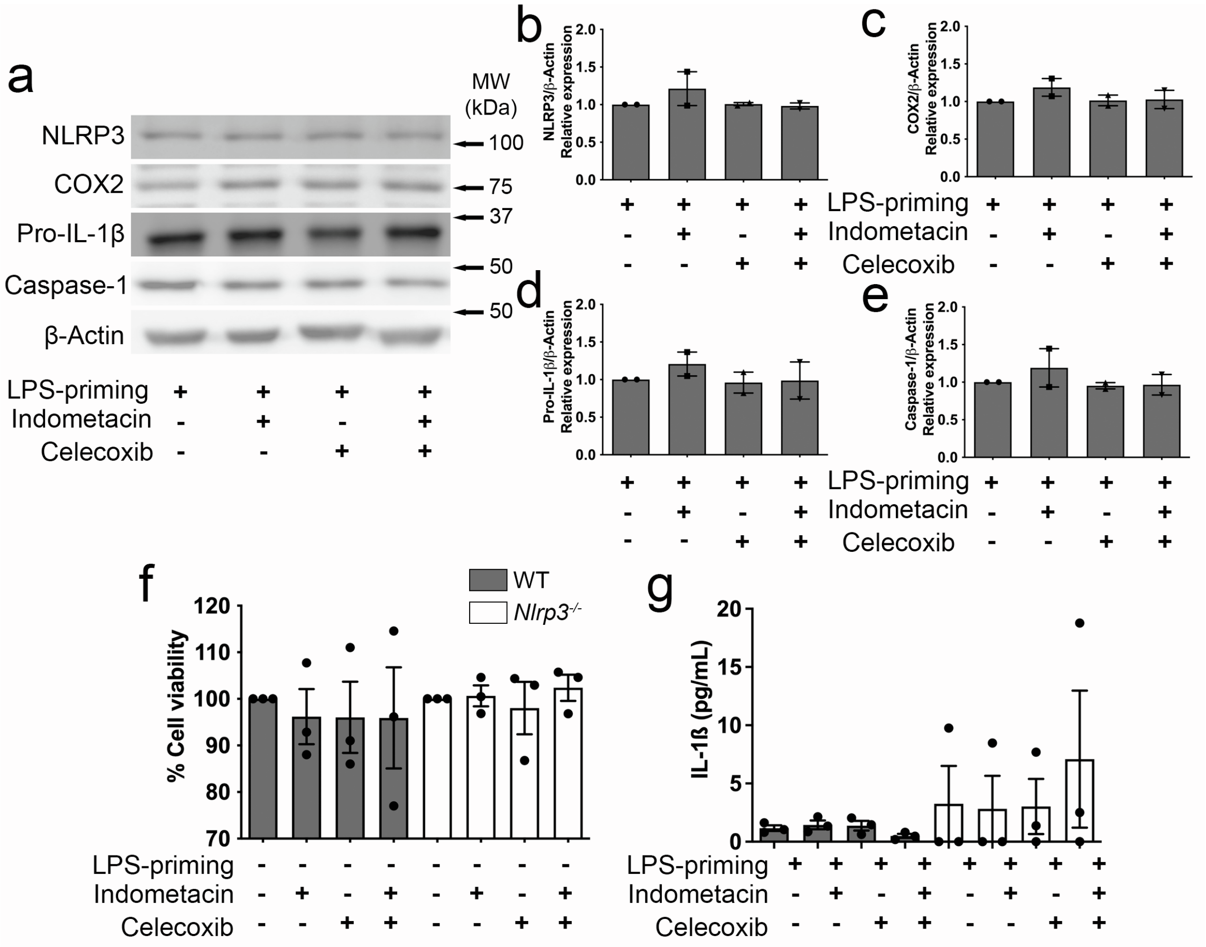

Figure S5: COX inhibition does not impact the expression of NLRP3-related proteins. (a-e) Expression and β-Actin-adjusted relative densitometry of NLRP3 (a, b), COX-2 (a, c), pro-IL-1β (a, d), and caspase-1 (a, e). (f-g) Cellular viability (f) and IL-1β production (g) in LPS-primed (200 ng/mL, 3 hours) WT and *Nlrp3*$^{-/-}$ BMDMs during one-hour incubation with 100 µM indomethacin, 10 µM celecoxib, or both. Dashed line represents the assay detection limit. (a) Image is representative of three independent experiments. (b-g) Data from three independent experiments (mean and s.e.m.).

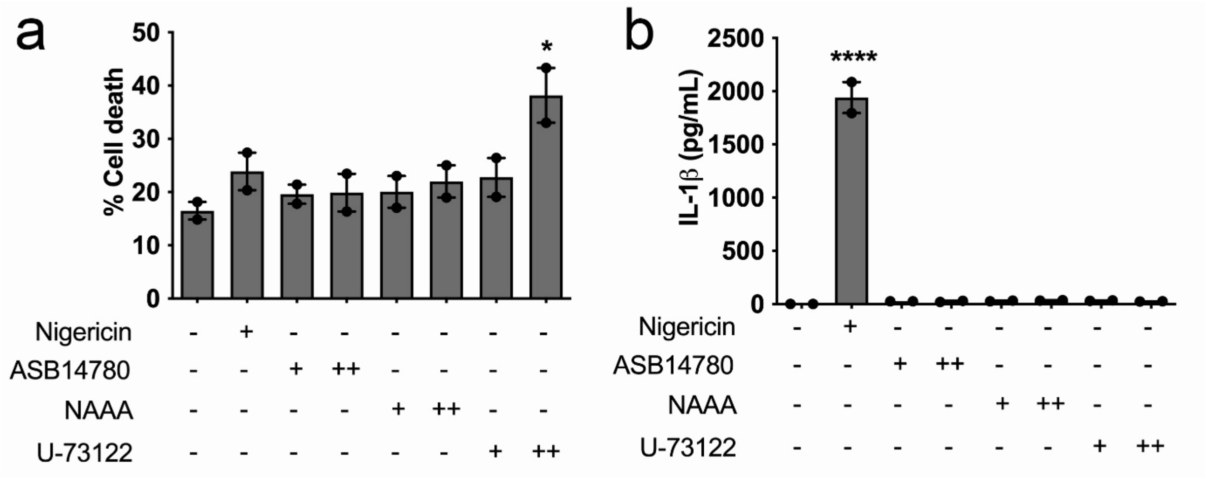

Figure S6: Effects of PLA_2 and PLC inhibitors on cell death and IL-1β production, related to Fig. 6. Cell death (a) and IL-1β production (b) in LPS-primed (200 ng/mL for 3 hours) WT BMDMs in response one-hour stimulation with 1 μM (+) or 10 μM (++) of PLA_2 inhibitors ASB1414780 and NAAA, or PLC inhibitor U-73122. * $p < 0.05$, **** $p < 0.0001$ in comparison to unstimulated control (one-way analysis of variance with Tukey's multiple comparison test). Data from two independent experiments (mean and s.e.m.).
